# Genome-wide investigation of the *dmrt* gene family reveals new insight into the gonad development in *Plectropomus leopardus*: *dmrt2a* regulate the development of oocytes

**DOI:** 10.1186/s13293-025-00769-6

**Published:** 2025-10-29

**Authors:** Hui Ding, Peiyu Li, Jingwen Wang, Chenlin Yin, Jiayi Wu, Jiahang Li, Qingran Guo, Zhenmin Bao, Bo Wang, Jingjie Hu

**Affiliations:** 1 MOE Key Laboratory of Marine Genetics and Breeding, College of Marine Life Sciences/Key Laboratory of Tropical Aquatic Germplasm of Hainan Province, Sanya Oceanographic Institution, Ocean University of China, Qingdao, Sanya China; 2 Southern Marine Science and Engineer Guangdong Laboratory, Guangzhou, 511458 China

**Keywords:** *Plectropomus leopardus*, *dmrt* gene family, *Dmrt2a*, RNAi, Oocyte development

## Abstract

**Background:**

*Plectropomus leopardus* is a hermaphrodite fish with a unique pattern of gonadal development. However, the molecular mechanism of sexual differentiation in this species remains unclear. The Doublesex and Mab-3 related transcription factor (*dmrt*) gene family are known to play a crucial role in gonad differentiation and development. Notably, systematic investigations into the composition and function of the *dmrt* gene family in this hermaphrodite fish remain conspicuously lacking.

**Methods:**

In this study, we systematically identified members of the *dmrt* gene family through genomic database mining in *P. leopardus*. Tissue and stage-specific expression profiles of *dmrt* paralogs were quantitatively analyzed using reverse transcription quantitative PCR (qPCR), revealing sexually dimorphic expression patterns in the gonads at various developmental stages. Furthermore, the expression distribution of *dmrt2a* at different developmental stages was explored using fluorescence in situ hybridization (FISH). Subsequently, *dmrt2a* was interfered with using RNAi technology, and the regulatory effect of *dmrt2a* on oocytes was verified by combining FISH and TUNEL assays.

**Results:**

In this study, we identified six members of the *dmrt* gene family in *P. leopardus* and designated them as *dmrt1*, *dmrt2a*, *dmrt2b*, *dmrt3*, *dmrta1* and *dmrta2* based on the homology analysis results, respectively. Whole-tissue expression analysis revealed that the *dmrt* genes exhibit tissue-specific expression pattern in *P. leopardus*. Notably, *dmrt1* and *dmrt2a* are highly expressed in the gonads, suggesting their potential role in gonadal development. Further qPCR results showed that *dmrt* genes are differentially expressed between males and females at different developmental stages. Among them, *dmrt2a* is highly expressed in the ovary at different developmental stages and is found to be a pivotal factor in ovarian development. FISH was used to further verify the expression of *dmrt2a* in oocytes. In addition, knockdown of *dmrt2a* in gonads caused oocyte apoptosis and decreased oocyte number, demonstrating the critical role of *dmrt2a* in oocyte development.

**Conclusions:**

This study demonstrates that *dmrt2a* plays a crucial regulatory role in the development of the oocytes in *P. leopardus*, supplementing the understanding of the functional roles of the *dmrt* gene family in vertebrate sex differentiation. These findings will help to understand the properties and functions of the *dmrt* genes in *P. leopardus* and provide a solid basis for further studies on the functional mechanisms of *dmrt* genes in hermaphroditic fish.

**Supplementary Information:**

The online version contains supplementary material available at 10.1186/s13293-025-00769-6.

## Introduction

Teleost account for nearly half of all extant vertebrates and exhibit a diverse array of sex determination mechanisms [1]. The mechanisms of sex determination in vertebrates are highly variable. While the primary sex-determining signals vary among species, many genes downstream of these signals exhibit a high degree of conservation [2]. Many genes involved in sex differentiation have been identified in vertebrates [3–6], such as *amhy* in *Oreochromis niloticus* and *Esox lucius*, *gdf6Y* in *Oryzias latipes*, and *dmrt1* in *Cynoglossus semilaevis* [7–10]. Despite the remarkable diversity of master sex-determining genes across species, the functional roles of the *dmrt* (Doublesex and Mab-3 related transcription factors) gene family in sex determination and differentiation remain evolutionarily conserved throughout the vertebrates [11]. The *dmrt* family genes are evolutionary derivatives of doublesex (*dsx*) in *Drosophila melanogaster* and male abnormal (*mab-3*) in *Caenorhabditis elegans* [12]. They constitute the *dmrt* gene family, a class of molecules characterized by a DNA-binding motif known as the DM domain [13]. The DM domain gene *dsx* was originally discovered from the fruit fly *Drosophila melanogaster* as a major sex regulatory gene, and was subsequently found to control sexual differentiation by producing alternatively spliced sex-specific mRNAs [3]. Another DM domain gene *mab-3* was identified in the nematode *Caenorhabditis elegans* [14]. Both the *mab-3* and *dsx* encoded proteins bind to similar DNA regulatory sequences of the control region of yolk protein genes and control their transcription [15]. Additionally, some *dmrt* genes have an additional conserved region termed the DMA domain, although the exact molecular mechanisms and functional significance of this domain remain unresolved [16, 17]. Currently, in addition to *dsx* and *mab-3*, the *dmrt* family in vertebrates include 9 *dmrt* genes (*dmrt1-8* and *dmrt2b*) that share common characteristics with *dsx* and *mab-3* [18].

*Dmrt* genes have been the focus of extensive scientific investigation due to their pivotal role as master regulators in both sex determination and differentiation pathways. For example, *dmrt1* is required for testis determination in the chicken [19], and a W-linked DM-domain gene, DM-W, participates in primary ovary development in *Xenopus laevis* [20]. This paradigm extends to aquatic vertebrates. In the *Oryzias latipes*, a Y-specific DMY gene, as a copy of autosome *dmrt1*, was found to be the master sex-determining gene inducing male formation. In general, given the key role of the *dmrt* gene in sex determination/differentiation, *dmrt* genes have been intensively investigated [20]. In vertebrates, *dmrt* genes have been identified in a wide range of species, including mammals, birds, reptiles, amphibians, and teleosts [21–23]. Seven *dmrt* genes have been found in fish, including *dmrt1*-*dmrt6* and *dmrt2b* [24, 25]. The *dmrt1*-*dmrt5* genes were relatively conserved in fish, while the dmrt6 gene was lost in most species during evolution [25]. The genomes of amphibians, re*ptil*es, birds, and mammals contain only a single *dmrt2* gene, whereas fish harbor two *dmrt2* genes (*dmrt2a* and *dmrt2b*). *dmrt2* is widely distributed in mammalian and fish tissues and is expressed in the testis and ovary. However, the function of *dmrt2* is not conserved across species evolution. Among them, *dmrt2a* is required for symmetrical somite formation and somogenesis, and *dmrt2b* is associated with slow development of branchial arches and muscles [26, 27]. This suggests that there are differences in the expression and function of *dmrt2a* and *dmrt2b* in fish. In addition, *dmrt3*, a member of the *dmrt* family, may be involved in testicular differentiation and development. The *dmrt4* gene can regulate the formation and development of ovarian follicles [28]. *Dmrt5* can regulate corticotropin and gonadotropin differentiation in the pituitary [29]. The functional diversification of *dmrt* paralogs across vertebrates highlights the dynamic interplay between evolutionary conservation and lineage-specific adaptation. Notably, gene duplication events, such as the retention of *dmrt2a* and *dmrt2b* in teleosts, have enabled subfunctionalization or neofunctionalization, allowing these paralogs to acquire distinct roles in gonadal development. For instance, *dmrt2a* is mainly expressed in male and female gonads or gills in adult fish. Exclusively, female expression has been identified in the gonad of the Atlantic cod, while *dmrt2b* regulates somite differentiation impacting on slow muscle development [30]. In most species where *dmrt* genes have been identified, however, the role of such genes in sex determination or differentiation remains unknown.

With the development of aquaculture, healthy gametes are a prerequisite for the reproduction of aquatic animals. Ovarian development is a key physiological process of great significance in fish reproduction, which determines the production of high-quality fertilized eggs. It has been shown that oogenesis is regulated by transcription factors, and several members of the transforming growth factor β (TGF-β) superfamily as well as transcription factors such as *bmp15*, *gdf9* and *foxo3* have been shown to play a role in different processes during oogenesis [31]. This suggests the important role of transcription factors in the regulation of oogenesis. The *dmrt* gene family, as a group of highly conserved transcription factors, also plays a central role in gonadal development. For example, *dmrt* family members can bind to different nuclear hormone receptors, activate receptor gene expression, and regulate estrogen levels [28]. Although members of the *dmrt* gene family have been studied in several species, their functional differences among different species still exist. Ovarian development is a complex process, and the specific functions of *dmrt* gene family members in this process are still unclear.

The leopard coral grouper (*Plectropomus leopardus*), which belongs to the *P. leopardus* genus of the *Epinephelidae* family [32], has become the most popular grouper species for aquaculture due to its attractive body coloration [33]. In our previous study, we found that the gonad development pattern of the *P. leopardus* is different from that of other fish, which makes the mechanism of sexual differentiation more complicated [34]. In previous studies, we have identified a series of genes involved in sex differentiation, such as *dmrt1*, *dmrt2a*, *sox3*, and *hsd17b12a*, but the roles of most of these genes remain unclear [35–37]. In this study, the primary goals were to carry out a genome-wide identification of the *dmrt* gene family in *P. leopardus*, and further explore the role of *dmrt2a* in oocyte development. Subsequent analysis demonstrated that *dmrt2a* gene could be a crucial factor in oocyte development of *P. leopardus*. Which could influence the development of ovary by regulating the apoptosis of oocytes. These studies will provide critical genomic resources for future studies on teleost *dmrt* genes and obtain a better understanding of their roles in sex differentiation and gonad development in *P. leopardus*.

## Materials and methods

### Sample collection and preparation

All samples used in this study were obtained from Hainan Chen Hai Aquatic Co., Ltd. (Hainan Province, China). At least nine healthy individuals were collected at different periods, namely, 12 months old, 15 months old, 2 years old, and 3 years old, with the aim of confirming the respective development stages of the specimens based on their histological morphology. At the same time, three healthy and similar size 9 months old *P. leopardus* were randomly selected for sampling. All samples were anesthetized with tricaine methanesulfonate (Sigma-Aldrich), and the gonad, skin, muscle, spleen, kidney, heart, liver, intestine, gill, and brain samples were collected. Half of each sample was immediately immersed in liquid nitrogen for RNA extraction, and the other half was fixed in 4% paraformaldehyde (Boster Biological Technology, USA) overnight. The gonads were dehydrated with serial ethanol (30%, 50%, 70%, 80%, and 90%) and stored in 100% ethanol for paraffin sectioning and fluorescence in situ hybridization (FISH).

### Gonadal histology and oocyte counting

The gonads were cleared in xylene, embedded in paraffin wax, and cut into 5–8 μm thick sections on a rotary microtome (Leica, Wetzlar, Germany). Serial sections were tiled on glass slides, deparaffinized with xylene, hydrated with graded ethanol to water, and stained with hematoxylin. Afterward, the glass slides were counterstained with eosin, dehydrated with ethanol, cleared with xylene, mounted with neutral balsam, and covered with coverslips. The sections were then observed and photographed under an Olympus BX43 microscope (Tokyo, Japan). Further analysis was conducted on the images of the gonads after RNAi. Oocytes were quantified in serial sagittal Sects. (5–6 μm thickness) of the entire ovary stained with hematoxylin and eosin (H&E). Every section was imaged at 10× magnification using Olympus BX43 microscope. Oocytes were counted manually in each section using ImageJ (https://imagej.net/ij/) cell-counter plugin (only oocytes containing a visible nucleus were counted) [38].

### Quantitative real-time PCR (qPCR) validation and statistical analysis

Total RNA was isolated from tissues using TRIzol (Invitrogen, Carlsbad, CA, USA) according to the manufacturer instruction [39], followed by DNase I (TaKaRa, Dalian, China) to remove DNA contamination. RNA quality and purity were assessed by 1.5% agarose gel electrophoresis and verified using an ultraviolet spectrophotometer (NanoDrop One, Thermo, Waltham, USA) with an A260/A280 ratio between 1.8 and 2.0. The expression profiles of *dmrt* genes were validated through qPCR analysis. The primers used in this study were designed using Primer Premier 5.0 based on the sequences of the genes tested, and the details of the primers are shown in Table S1. The qPCR was performed on a Bio-Rad CFX96 Real-Time PCR System following the manufacturer standardized protocol. Amplifications were carried out using BlasTaq 2X qPCR MasterMix (Applied Biological Materials, Canada) with cycling conditions optimized according to the established methodology described by previously method [40]. *B2m* was used as the reference gene to normalize the expression in various tissues and different stages gonad according to our previous validation [40–47]. The relative expression levels were calculated by the 2^−ΔΔCt^ method [48]. All data are expressed as the mean ± SD of three replicates.

### Identification and structural features of the dmrt gene family

To ascertain the members of the *dmrt* gene family in *P. leopardus*, the representative amino acid sequences of *dmrt* gene family of other species were downloaded from the NCBI genome database (https://www.ncbi.nlm.nih.gov/genome) and Ensembl database (https://asia.ensembl.org/index.html) to construct a local database. The genomes of *P. leopardus* were obtained from NCBI (NCBI: PRJNA545594) [41]. Representative amino acid sequences of the DMRT gene family from other species were compared with the *P. leopardus* genome database through local BLASTP search (https://blast.ncbi.nlm.nih.gov/Blast.cgi) with the *E*-value set at 1e^−5^ [49]. Members of the *P. leopardus* DMRT gene family were identified through this BLAST analysis. The names of the tagged genes could be used directly, and the unnamed genes were named according to other teleost fishes. The chromosome distribution of the *dmrt* genes was predicted by TBtools [50], based on genomic and Genome annotation data [41]. Expasy software (https://web.expasy.org/) was used to predict the ORF length, amino acid number, molecular weight (D), isoelectric point (pI), and other structural characteristics of *dmrt* gene family members [51].

### Structure and motif analysis of DMRT gene family proteins

The SMART (https://smart.embl.de/) was used to predict the functional domains of the protein [52]. The online software IBS was used to visualize the protein domain characteristics of the *dmrt* gene family [53]. Motif analysis of conserved sequences of DMRT proteins was performed using MEME online software (MEME-submission form (meme-suite.org)) [54]. The program was set as follows: the number of motifs was set as 5, and the width was set as 6–50.

### Phylogenetic analysis of the dmrt gene family

The full-length amino acid sequences of *dmrt* genes derived from 16 species were used for phylogenetic analysis (Supplementary S1). To investigate the phylogenetic relationships between the *dmrt* gene family of *P. leopardus* and other species, a phylogenetic tree was constructed by MEGA11 using DMRT amino acid sequences, based on the neighbor-joining (NJ) method, and the bootstrap was set to 1000 [55]. Finally, the constructed phylogenetic tree was modified by the online tools iTOL (http://itol.embl.de/) [56].

### Analysis of dmrt genes expression based on transcriptome database

To investigate the function of the *dmrt* family in gonadal development, we performed an extensive analysis of the expression levels of *dmrt* gene in transcriptome libraries at different stages of gonadal development. Illumina data were obtained from our previous study [34]. Clean reads were mapped to a reference genome of *P. leopardus* (NCBI: PRJNA545594) by Hisat2 (v2.1.0) (https://daehwankimlab.github.io/hisat2/) [57]. FeatureCounts (http://subread.sourceforge.net) was used to count the number of reads mapped to each gene [58]. The abundances and variations in gene expression were calculated and normalized using transcripts per kilobase of exon per million mapped reads. Then, the expression of *dmrt gene* in gonads at different developmental stages was derived from the transcriptome data. Weighted gene co-expression network analysis (WGCNA) was performed using the WGCNA R package [59] based on normalized gene expression data obtained from 15 tissue transcriptome datasets. Gene ontology (GO) enrichment and KEGG pathway analyses were performed using OmicShare tools (www.omicshare.com/tools).

### Knock down of dmrt2a by DsRNA injection

BLOCK-iT™ RNAi Designer (https://rnaidesigner.thermofisher.com/rnaiexpress/) to predict three dsRNAs sequences, and synthesized by Sangon Biotech (Sangon, China). After synthesis, the knockdown efficiency of the three dsRNAs was detected. At the stage of sex differentiation, the three dsRNAs were intraperitoneally injected at a dose of 0.5 µg per gram of fish body weight (0.5 µg/g) into 9 healthy *P. leopardus* (210dph) [60]. The three controls were injected with the same volume of control dsRNA and cell transfection reagent mixture. After 48 h of anesthesia, gonadal samples were collected for RNA extraction and qPCR analysis of *dmrt2a* expression, and the dsRNA site with the highest knockdown efficiency was selected for subsequent experiments. Intraperitoneal injections were performed at weekly intervals for a total of four times at the same dose as in the pre-experiment (0.5 µg/g). A sample of gonads was collected on the 7th day after the last injection was completed. The gonads were divided into two parts, one part was preserved in RNAwait solution for subsequent RNA extraction, and the other part was preserved in PFA solution for subsequent histological observation, FISH and TUNEL cell apoptosis detection.

### Dual-color fluorescence in situ hybridization

The localization of *vasa*, *dmrt2a* and *zp4* was analysed by FISH. The selection of *vasa* and *zp4* mRNA probes was based on previous studies and unpublished data [61]. *Vasa* has been confirmed through our previous research to be a marker gene for germ cells in *P. leopardus* [61]. *Zp4* has been confirmed based on our unpublished data to be a potential marker gene for oocytes. Moreover, in other species, *zp4* has been proven to be an oocyte-specific marker gene for *Oryzias latipes* [62], *Haliotis discus hannai* [63], and *Coturnix japonica* [64]. FISH was performed using previously modified method [34, 65]. cDNA fragments avoiding conserved DNA binding domains were amplified with specific primers containing a T7 or SP6 promoter sequence (the primers are listed in Table S2) to ensure the specificity of the probe. The product of polymerase chain reaction (PCR) was inserted into the pMD^TM^-19T Vector (TaKaRa, Shiga, Japan) and sequenced to ensure the accuracy of the product sequences. These cloned products served as templates for in vitro transcription using T7 or SP6 RNA polymerase to generate sense and antisense RNA probes. Probes for *vasa* were synthesized using the DIG RNA Labeling Mix (Roche, Mannheim, Germany), while probes for *dmrt2a* and *zp4* were synthesized using both the DIG RNA Labeling Mix and the Biotin RNA Labeling Mix, respectively (Roche, Mannheim, Germany). The gonadal tissues from four developmental stages were cut into 6-µm-thick sections on glass slides coated with 0.1% poly-L-lysine solution. After dewaxing and rehydration, the gonadal sections were subjected to PBS treatments containing 0.1% Tween-20 (PBST) for 10 min and digested with 2 µg/mL of proteinase K at 37 °C for 15 min. Afterward, the gonadal sections were prehybridized at 56 °C for 3 h. The probes were subjected to denatured incubation at 95 °C for 5 min then hybridized by a 1 ng/µL denatured probe at 56 °C for 17 h. Subsequently, the slides were washed in SSC. The tissue sections were incubated with blocking-1 at 37 °C for 30 min and incorporated with anti-digoxigenin–fluorescein and Streptavidin − Cy3™ (from Streptomyces avidinii) at a ratio of 1:200 in blocking-1 for 1 h at 37 °C to detect the hybridization signal. Later, the sections were counterstained with DAPI for cell nuclear staining to confirm the number and status of germ cells. Afterward, images were acquired with an Olympus FV3000 confocal microscope (Olympus, Japan) using the FV31S-SW software.

### TUNEL assays

For terminal deoxynucleotidyl transferase dUTP nick-end labeling (TUNEL) assays, DNA damage analysis was performed using a Colorimetric TUNEL Apoptosis Assay Kit (Beyotime, China). Briefly, after deparaffinized with xylene, the gonads Sects. (5–6 μm) were rehydrated and permeabilized with 20 µg/mL of proteinase K for 15 min. Then, the samples were inactivated with enhanced endogenous peroxidase blocking buffer for 20 min. Samples were incubated with the TUNEL reaction mixture at 37 °C for 60 min and were stained with streptavidin-HRP at room temperature for 30 min. The sections were counterstained with hematoxylin after TUNEL staining. Afterward, TUNEL-positive cells were brown, and nuclei were stained as blue [66]. The sections were then observed and photographed under an Olympus BX43 microscope (Tokyo, Japan).

## Results

### Identification and sequence analysis of dmrt genes in P. leopardus

A total of 6 members of the *dmrt* gene family were identified from *P. leopardus* with bioinformatics analysis based on its genomic information and designated as *dmrt1*, *dmrt2a*, *dmrt2b*, *dmrt3*, *dmrta1*, *dmrta2*. The sequence information for the *dmrt* family of *P. leopardus* is presented in Table 1. The predicted relative molecular weights ranged from 31949.81D (*dmrt1*) to 76734.38D (*dmrta1*), with protein lengths ranging from 294 (*dmrt1*) to 694 amino acids (*dmrta1*). And isoelectric points ranged from 5.91 to 9.19. The six genes of the *dmrt* gene family are distributed on three chromosomes, with *dmrt1*, *dmrt2a* and *dmrt3* were located on chromosome 6, *dmrt2b* and *dmrta2* were on chromosome 3, and *dmrta1* was on chromosome 23. Detailed information of the *dmrt* genes is shown in Table 1.

**Table 1:** The detailed information of *P. leopardus* DMRT proteins

Gene name	Location	ORF length	Amino acids number	Molecular weight (Da)	Isoelectric points (pl)
*dmrt1*	Chr6:32452395–32,486,964	885	294	31949.81	8.75
*dmrt2a*	Chr6:32510129–32,515,705	1569	522	57096.48	8.79
*dmrt2b*	Chr3:11978087–11,982,226	1341	446	50029.88	9.19
*dmrt3*	Chr6:32496600–32,500,491	1413	470	51090.12	5.91
*dmrta1*	Chr23:11987968–11,989,868	1263	694	76734.38	6.73
*dmrta2*	Chr3:23495560–23,510,944	1344	447	47577.31	8.11

### Molecular characters of P. leopardus dmrt gene family

**Fig. 1:**
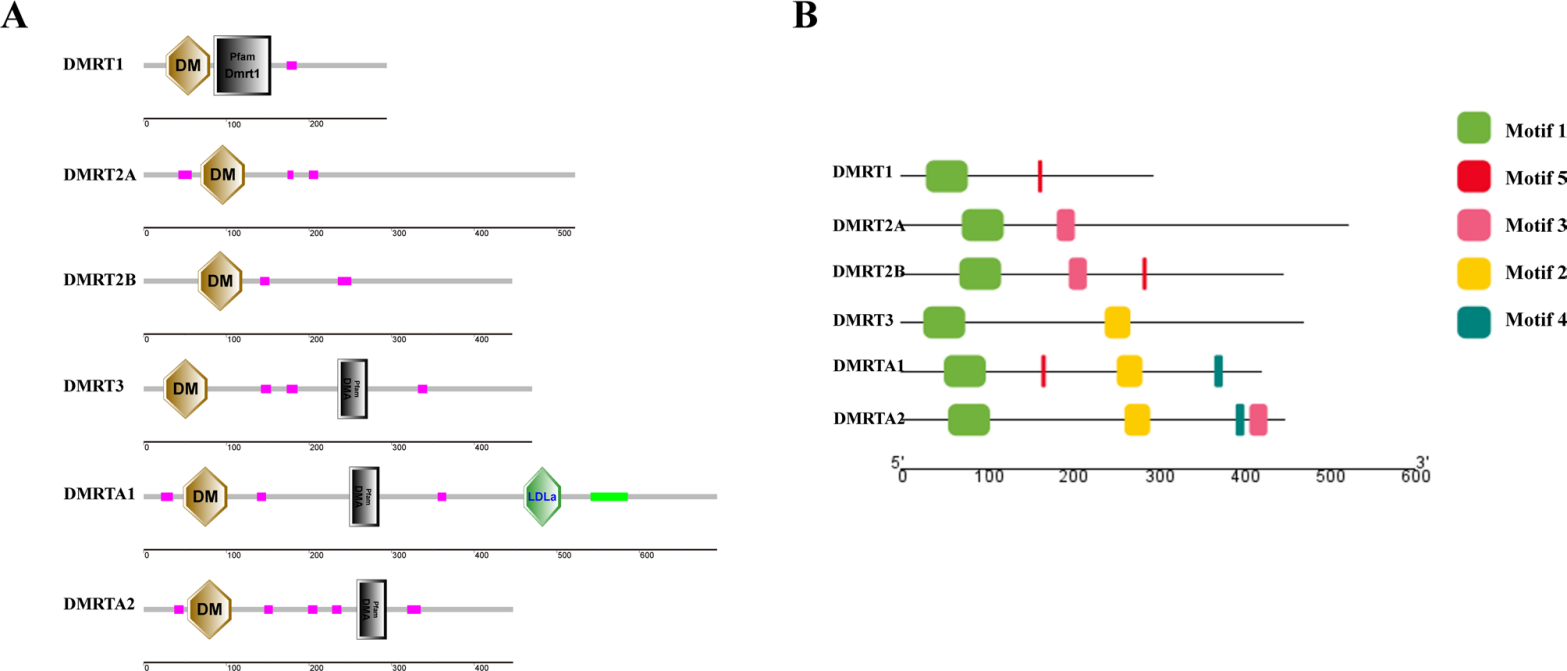
Molecular characteristics of the *dmrt* gene family in *P. leopardus*. (**A**) Domain architecture of DMRT proteins. (**B**) Motif analysis of the *dmrt* gene family

Figure 1A shows the protein domains of the *dmrt* gene family in *P. leopardus*. All six members possess a conserved DM domain, which is capable of binding DNA and plays a role in sex differentiation [67]. In addition to the DM domain, DMRT1 possesses the DMRT1 domain or DMRT1 superfamily domain as its main distinguishing feature [68, 69]. DMRT2A and DMRT2B only contained the DM domain. DMRT3, DMRTA1 and DMRTA2 have DMA domain located at the C-terminus of the DM domain within a region of 200 to 300 amino acids. In addition, DMRTA1 also has a type A low-density lipoprotein domain (LDLa). Figure 1B presents the motif predictions for the *P. leopardus dmrt* gene family. Each family member contains a Motif1, arranged in alignment with the predicted protein domain and likely corresponding to the DM domain. Additionally, DMRT3, DMRTA1, and DMRTA2 share a common Motif2, which is likely representative of the DMA domain. It is hypothesized that the DM and DMA structures in the DMRT family proteins have remained largely unchanged over evolutionary time, supporting the conservation of their functional roles.

### The phylogenetic analysis of dmrt gene family

**Fig. 2:**
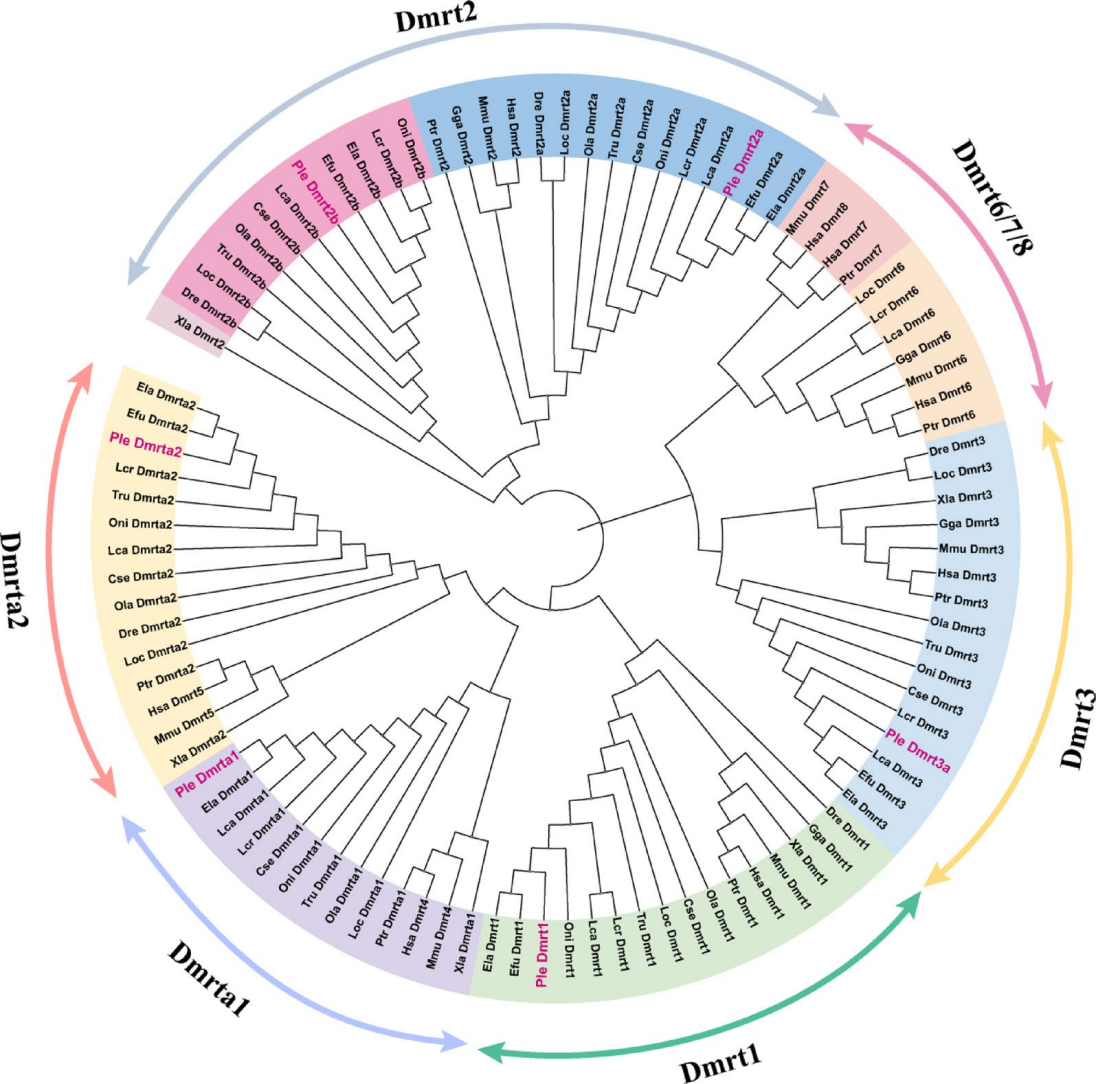
The phylogenetic analysis of the DMRT proteins with other known DMRT proteins. Note: Hsa: *Homo sapiens*, Mmu: *Mus musculus*, Dre: *Danio rerio*, Xla: *Xenopus laevis*, Gga: *Gallus gallus*, Ola: *Oryzias latipes*, Ptr: *Pan troglodytes*, Cse: *Cynoglossus semilaevis*, Loc: *Lepisosteus oculatus*, Tru: *Takifugu rubripes*, Lcr: *Larimichthys crocea*, Lca: *Lates calcarifer*, Oni: *Oreochromis niloticus*, Ple: *Plectropomus leopardus*, Efu: *Epinephelus fuscoguttatus*, Ela: *Epinephelus Lanceolatus*. The protein sequences used in this study were shown in the Supplement file

Phylogenetic analysis (Fig. 2) indicates that all *dmrt* genes in *P. leopardus* cluster with their orthologs in other species, suggesting that the *dmrt* gene family is highly conserved. The phylogenetic clustering reveals three major groups and eight subfamilies. Group 1 includes four subfamilies: *dmrt3*, *dmrt6*, *dmrt7*, and *dmrt8*. Only *dmrt3* is present in the *P. leopardus* genome, with no homologs of *dmrt6*, *dmrt7*, or *dmrt8* identified. *Dmrt7* and *dmrt8* are specific to mammals, with *dmrt8* being unique to humans. *Dmrt6* is absent in species outside mammals and some teleost fish. Group 2 includes three subfamilies: *dmrt1*, *dmrta1*, and *dmrta2*, suggesting closer evolutionary relationships among these subfamilies. Group 3 consists of a single subfamily, *dmrt2*, with *dmrt2a* and *dmrt2b* as two copies found only in teleosts, suggested that the evolutionary relationship between *dmrt2a* and *dmrt2b* is particularly close. Additionally, all *dmrt* family members in *P. leopardus* cluster with other teleost fish, consistent with its evolutionary position. Furthermore, synteny analysis indicated that duplicated *dmrt* genes were generated from teleost genome duplication events, the neighboring genes of which on the chromosome were relatively highly conserved among teleost species (Fig. S1).

### Expression patterns of dmrt genes in different tissues

**Fig. 3:**
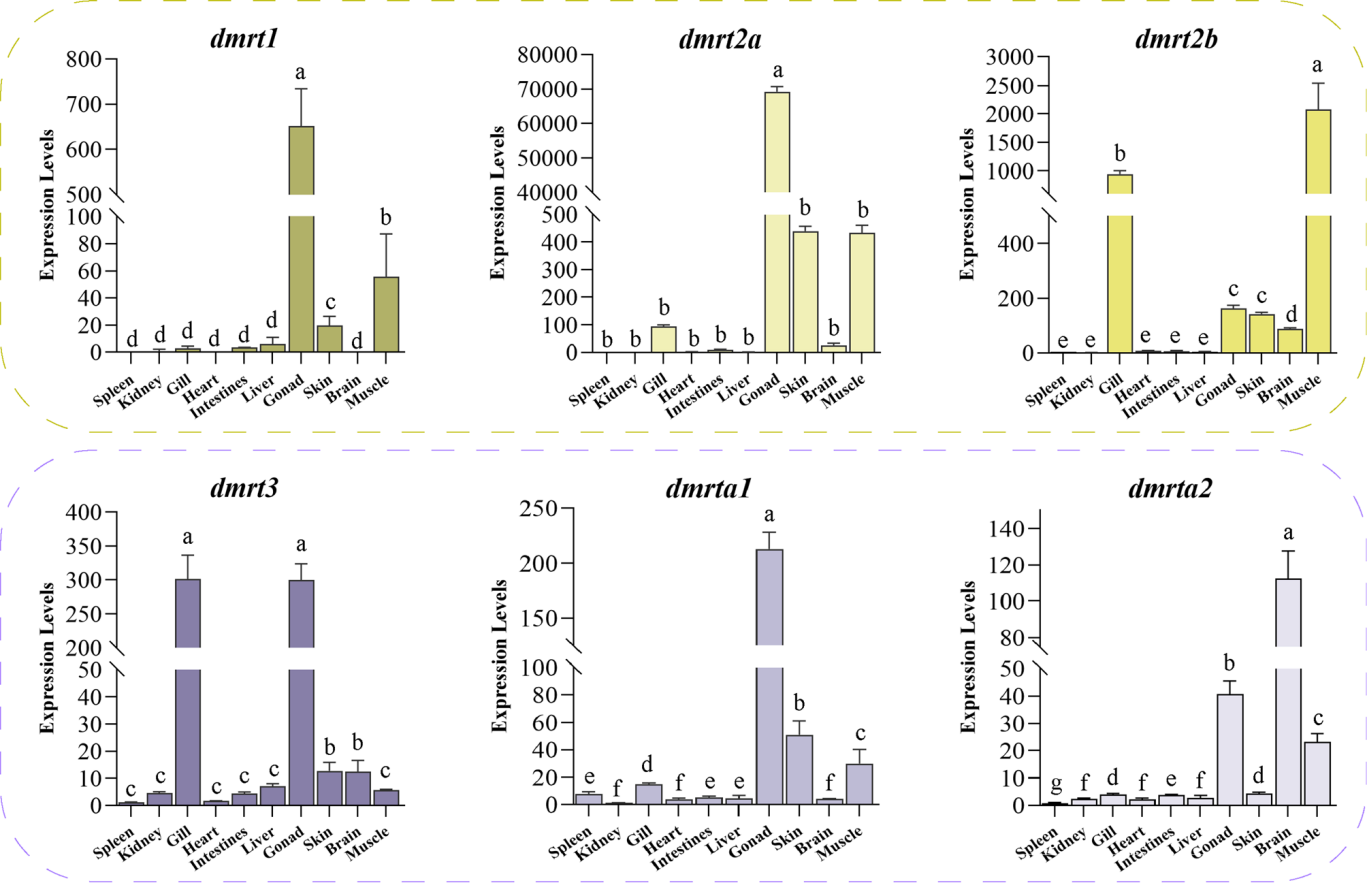
Expression levels of *dmrt* genes in various tissues of *P. leopardus*. Errors bars represent the standard error of means (means ± SE, *n* = 3)

The expression patterns of six *dmrt* genes in various tissues of *P. leopardus*, including skin, muscle, gill, spleen, heart, brain, intestine, liver, and gonad were analyzed by qPCR (Fig. 3). The results showed that the expression levels of *dmrt1* and *dmrt2a* genes were higher compared with other *dmrt* genes, and both genes were highly expressed only in gonad and hardly expressed in other tissues. *Dmrt2b* was highly expressed in gill and muscle, weakly expressed in gonad, and to some extent expressed in skin and brain. The expression of *dmrt3* was higher in gonad and gill, and lower in other tissues. The expression of *dmrta1* in gonad was significantly higher than that in other tissues and the expression of *dmrta2* in brain was the highest, followed by gonad. Most of the *dmrt* genes were mainly expressed in the gonad, suggesting that the *dmrt* gene family plays an important role in gonadal development of *P. leopardus*.

### Expression patterns of dmrt genes in gonads at different developmental stages

**Fig. 4:**
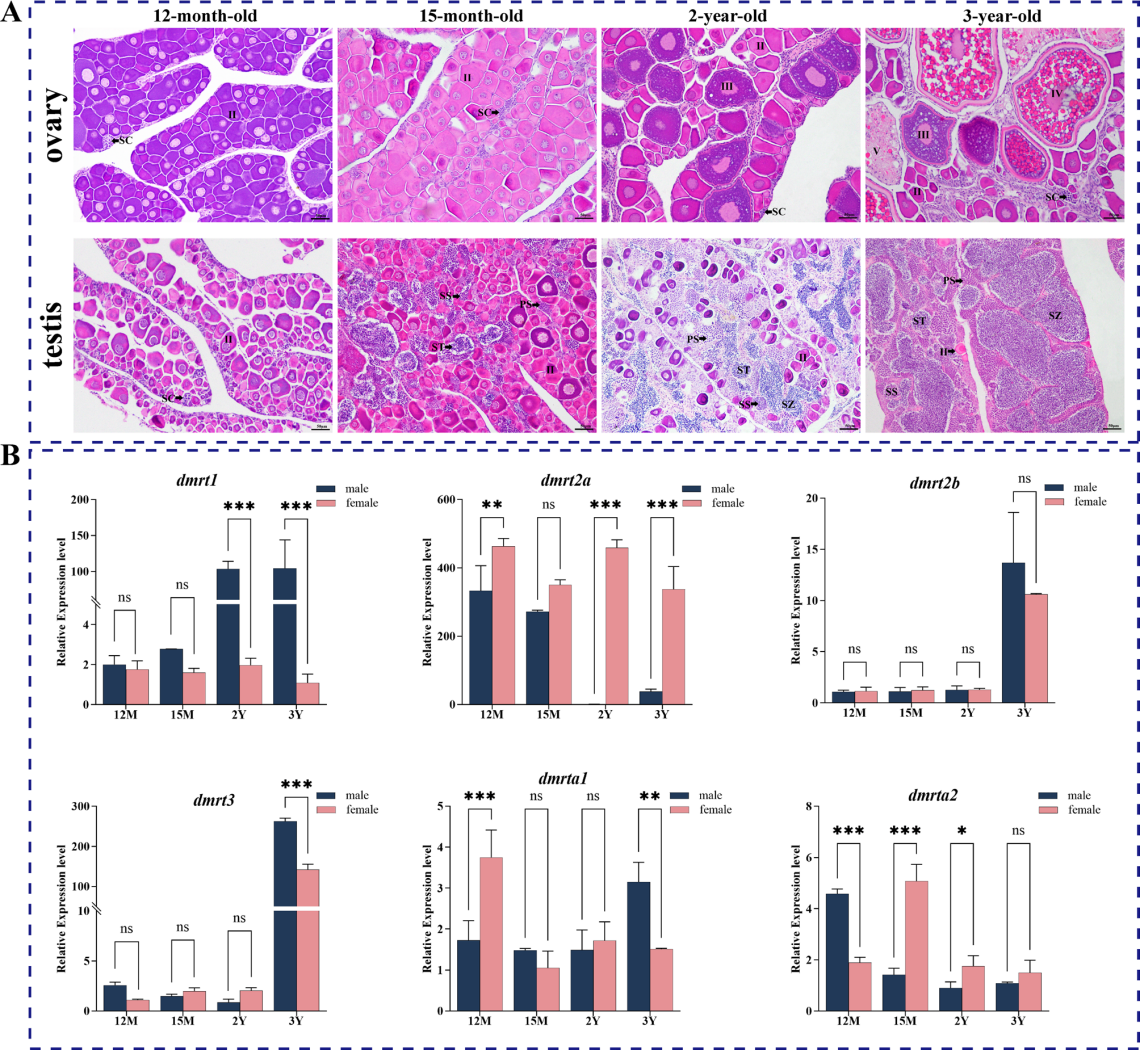
Expression patterns of the *dmrt* gene family in *P. leopardus* at different developmental stages. (**A**) Histological observation of the gonad of *P. leopardus* at different developmental stages. (**B**) Differential expression of the *dmrt* gene family. Ⅱ, primary stage oocyte; Ⅲ, cortical-alveolus stage oocyte; Ⅳ, vitellogenic stage oocyte; Ⅴ, atretic follicle stage oocytes; SC, spermatogenic cysts; PS, primary spermatocyte; SS, secondary spermatocyte; ST, spermatid; SZ, spermatozoa

Previous results indicate that members of the *dmrt* gene family exhibit high expression levels in the gonads. To investigate the spatiotemporal expression patterns of the *dmrt* gene family in gonads, histological observations were conducted on male and female gonads at different developmental stages (12-month-old, 15-month-old, 2-year-old, and 3-year-old). At the 12-month-old, gonadal sex differentiation has begun, with gonadal development diverging into ovarian development and testis development. During ovarian development, stage Ⅱ oocytes are predominant, with a gradual reduction in spermatogenic cysts. Oocytes development from stage II to mature oocytes at stages III and IV. In testicular development, the number of spermatogonia cysts increases, followed by the appearance of primary spermatocytes, secondary spermatocytes, spermatids, and spermatozoa (Fig. 4A).

Based on the histological observations, the expression patterns of *dmrt* gene family members in female and male gonads at different developmental stages were explored (Fig. 4B). The qPCR results showed that *dmrt1* has very low expression in the ovary at different developmental stages, with a low expression in the early testis (12-month-old and 15-month-old), followed by a significant increase and the highest expression in the mature testis, suggesting that *dmrt1* may promote the development and maturation of the testis. The expression levels of *dmrt2a* were consistently higher in the ovary than in the testis at all developmental stages, indicating that *dmrt2a* may facilitate ovarian differentiation and maturation. However, there was no significant difference in *dmrt2b* between male and female gonads. *Dmrt3* displayed marked differences in expression at gonadal maturation, with higher levels in males than in females. The expression of *dmrta1* was significantly different between male and female gonads at 12-month-old and 3-year-old, while the expression of *dmrta2* showed a different bias between male and female gonads at different developmental stages and gradually decreased with gonadal development. Notably, *dmrt1* and *dmrt2a* exhibit sex-biased expression patterns, with *dmrt2a* showing consistently higher expression levels during ovary development.

### Identification the key genes involved in oocyte development

**Fig. 5:**
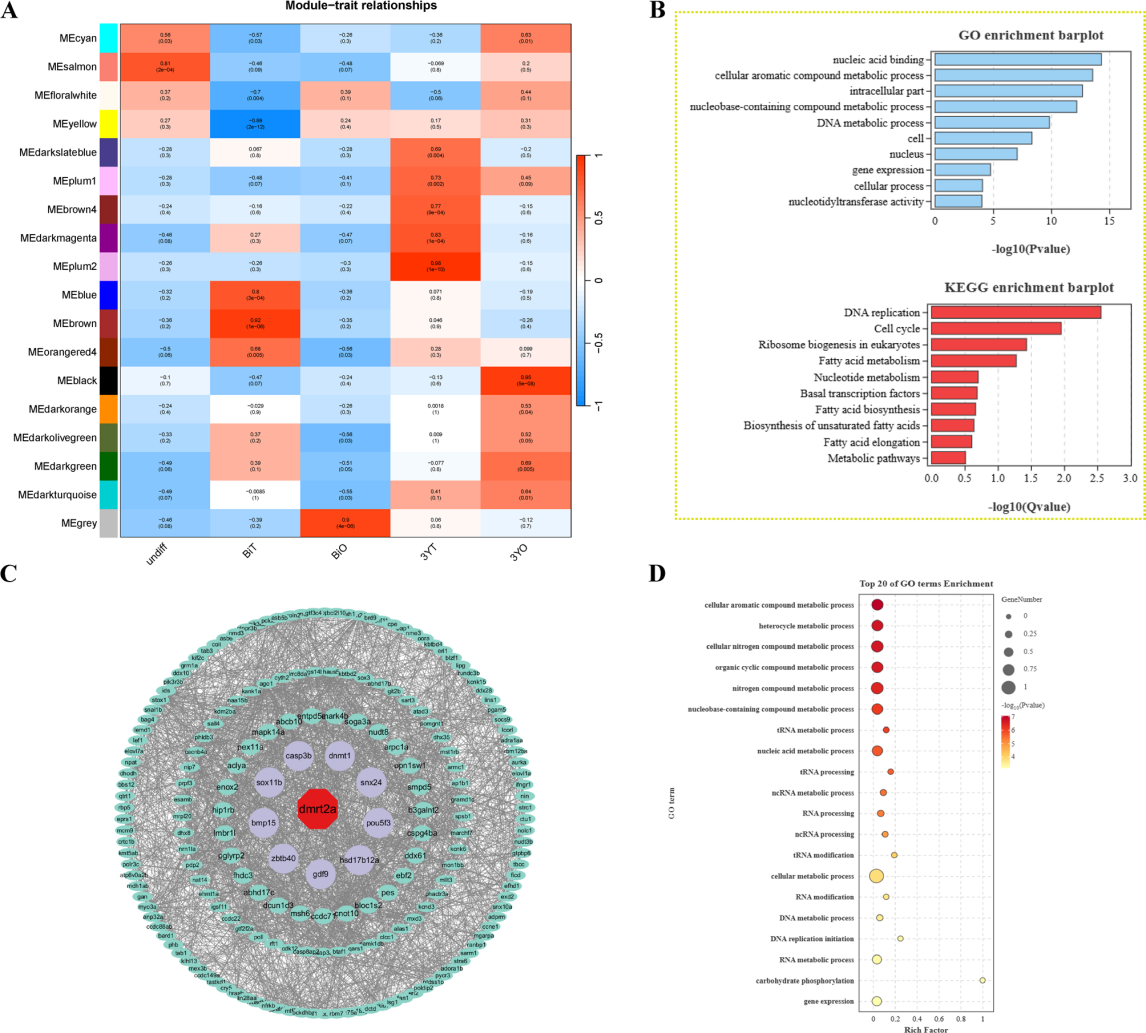
Weighted gene co-expression network analysis (WGCNA). (**A**) The relationship among 18 modules, where the colour bar indicates the correlation value from low (blue) to high (red). undiff, 120 days post-hatching (dph) gonad; BiT, 15 months old bisexual-phase (Bi) testis; BiO, 15 months old bisexual-phase (Bi) ovary; 3YT, 3 years old testis; 3YO, 3 years old ovary. (**B**) GO and KEGG analysis of genes in the black module. (**C**) The gene co-expression networks of black module. (**D**) GO enrichment of genes co-expressed with *dmrt2a*

To identify *dmrt* members associated with ovary development in the gonad, WGCNA analysis was performed using 15 transcriptome datasets from gonad at different developmental stages, which resulted in the generation of 18 gene modules. Among these, black module was found to be significantly related to the ovary development (Fig. 5A). Subsequently, GO and KEGG enrichment analysis was conducted using genes from black module. As shown in Fig. 5B, the genes from black module were significantly involved in nucleic acid binding, cellular aromatic compound metabolic process, basal transcription factors and fatty acid metabolism. This indicates that the module plays an important role in oocyte development and maturation. Further, gene co-expression networks with top 5% weighted correlations were constructed using annotated genes from the black module. We identified *dmrt* family member *dmrt2a* as a hub factor. GO enrichment analysis also revealed that the genes co-expressed with *dmrt2a* were all known to be involved in oocyte and ovarian development, such as *hsd17b12a*, *gdf9*, and *bmp15* (Fig. 5C, D).

### Dynamic changes of dmrt2a during male and female development

**Fig. 6:**
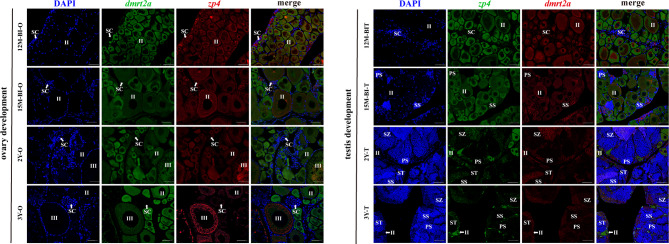
Co-localization of *dmrt2a* and *zp4* antisense probe in *P. leopardus*. Ⅱ, primary stage oocyte; Ⅲ, cortical-alveolus stage oocyte; SC, spermatogenic cysts; PS, primary spermatocyte; SS, secondary spermatocyte; ST, spermatid; SZ, spermatozoa; Scale bars = 50 μm

FISH results (Fig. 6) showed that during ovarian development, the fluorescence signals of *zp4* and *dmrt2a* were co-localized in oocytes at various developmental stages. No significant fluorescence signal of *dmrt2a* was found in SC, but its fluorescence signal gradually decreased with development in oocytes. During testis development, the co-localization of *dmrt2a* and *zp4* persisted. However, the fluorescence signal of *dmrt2a* in oocytes gradually diminished as testis development progressed. Notably, no *dmrt2a* fluorescence signal was detected in spermatocytes at all stages.

### The effect of dmrt2a on oocyte development in P. leopardus

**Fig. 7:**
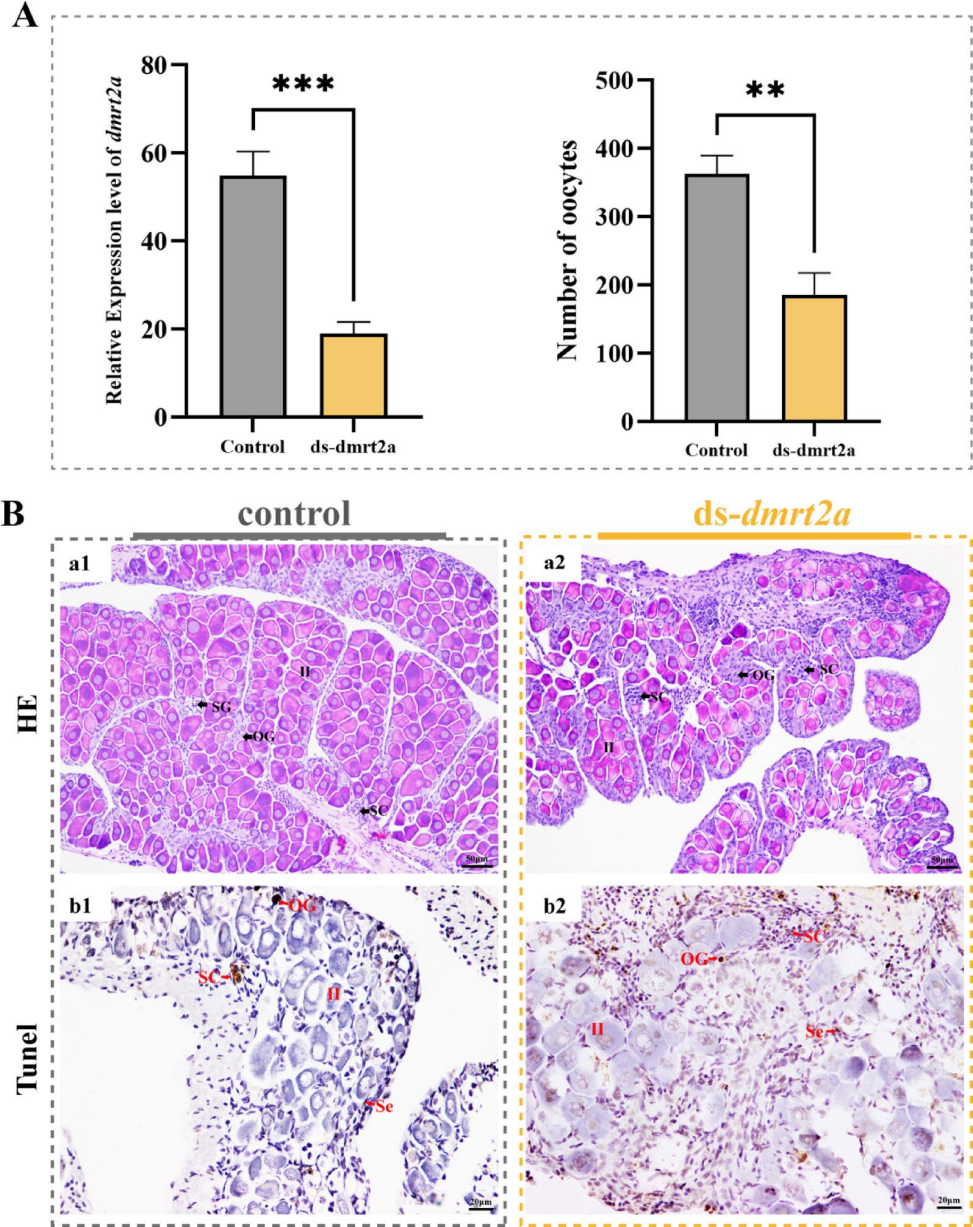
Function of *dmrt2a* in ovarian development of *P. leopardus*. (**A**) The relative expression levels of *dmrt2a* and number of oocytes in the gonad after RNAi experiment. Marks *** and ** indicated the statistical difference (*P* < 0.01 and *P* < 0.001). Data was displayed by mean ± SEM (*N* = 3). (**B**) Effect of knockdown on gonadal development. (a1, a2) Histological observation after RNAi interference. (b1, b2) Apoptosis signal locations in the control and experimental groups after RNAi interference. Brown cells indicate the location of the apoptotic signal. GSC, germ stem cell; OG, oogonia; Ⅱ, primary stage oocyte; SC, spermatogenic cysts; Se, sertoli cell

To explore the specific role of *dmrt2a* in oocyte development in *P. leopardus*,* dmrt2a* knockdown assays were performed in *P. leopardus* bass using dsRNA. Three dsRNA were examined for knockdown efficiency, and the one with the highest knockdown efficiency siRNA-1226 was selected for injection (Fig. S2). The expression of *dmrt2a* in ds-*dmrt2a* group was significantly lower than that in control group, indicating that knockdown would cause a significant decrease in *dmrt2a* expression (Fig. 7A). Histological results showed that the number of oocytes in ds-*dmrt2a* group was lower than that in the control group (Fig. 7A), and the oocytes were more loosely arranged in the gonad (Fig. 7B). In addition, TUNEL assay showed that apoptosis signals were mainly found in some SC and Se in the control group, but almost no apoptosis signals were detected in oocytes and GSC (Fig. 7C). In ds-*dmrt2a* group, there were more apoptotic signals in oocytes, oogonia and Se, but only partial apoptotic signals in SC. Therefore, the reduced number of oocytes in ds-*dmrt2a* group was inferred to be due to the apoptosis of oocytes caused by *dmrt2a* interference, further underscoring the critical role of *dmrt2a* in promoting oocyte development.

**Fig. 8:**
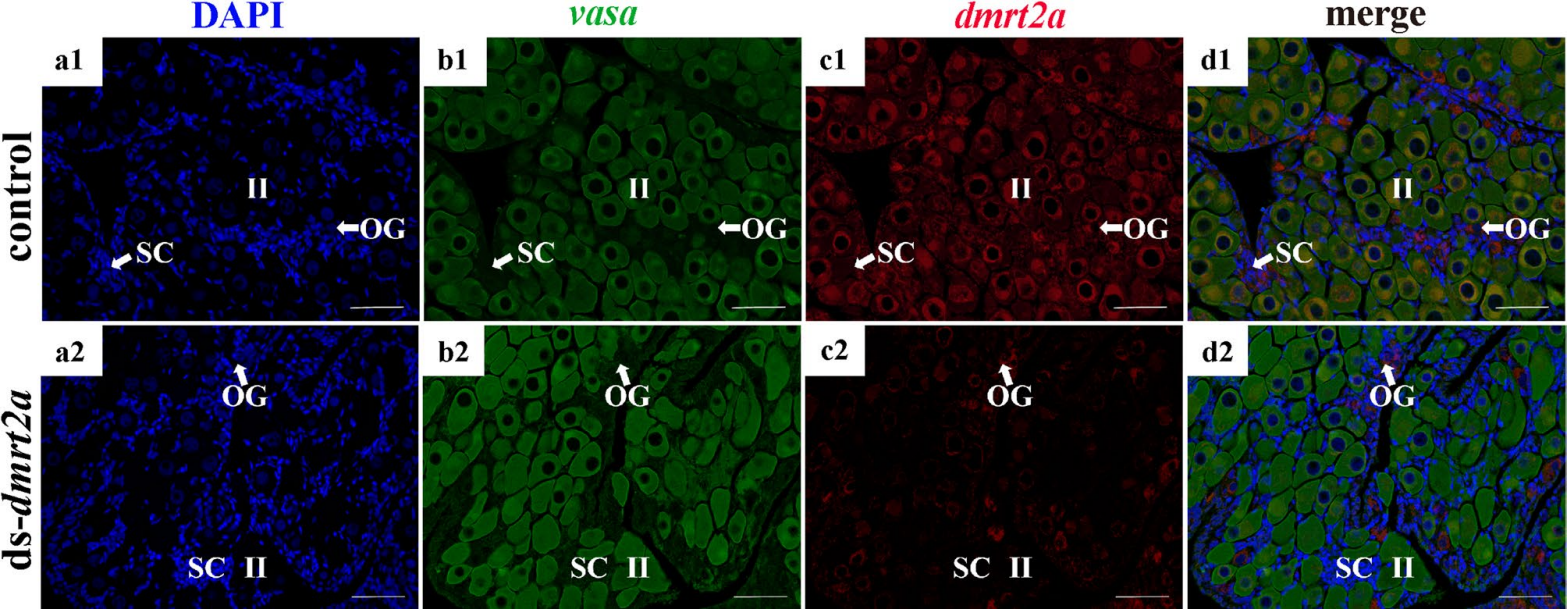
Co-localization of *dmrt2a* and *vasa* antisense probe in the control and experimental groups after RNAi interference. (a1-d1) co-localization of *dmrt2a* and *vasa* antisense probe in the control groups; (a2-d2) co-localization of *dmrt2a* and *vasa* antisense probe in the experimental groups. OG, oogonia; SC, spermatogenic cysts; Ⅱ, primary stage oocyte. Scale bars = 50 μm

To further explore the differences in the expression and localization of *dmrt2a* between the experimental and control groups FISH were performed (Fig. 8). The results showed that *vasa* labeled all germ cells in the control and experimental groups. In the control group, the fluorescence signal of *dmrt2a* was detected in stage II oocytes and oogonia, but not in spermatogenic cysts, which was similar to the previous results of *dmrt2a* localization in the gonad of *P. leopardus*. Notably, no signal was detected in the SC of the ds-*dmrt2a* group, and its signal distribution was reduced in stage II oocytes and oogonia (Fig. 8). Knockdown of *dmrt2a* reduced the signal in stage II oocytes and oogonia, indicating that *dmrt2a* plays a critical role in the oocyte development of *P. leopardus*.

## Discussion

Sex determination and differentiation in teleost is a complex process involving the regulation of multiple genes. The *dmrt* gene family has been extensively studied and is recognized as playing critical roles in embryonic sex differentiation, gonadal development, and gametogenesis in fish [21]. The *P. leopardus*, belong to the hermaphrodite fish, but the molecular mechanism of sexual differentiation in this species remains unclear. In this study, we systematically identified and characterized the *dmrt* gene family in *P. leopardus*. A total of six *dmrt* gene family members were identified in the whole genome, which were classified into different subfamilies based on conserved exon-intron structures and protein domains. Among them, the *dmrt1*-*dmrt3*-*dmrt2a* cluster was found tandemly arranged on chromosome 6, while *dmrta1* and *dmrta2* were located on different chromosomes. This genomic is consistent with the evolutionary history of teleost-specific genome duplications inferred for *dmrt* genes in teleosts [70]. As with most teleost, all six *dmrt* gene family members in *P. leopardus* contain a conserved traditional DNA-binding domains (DM domains), suggesting that the composition of the *dmrt* family in *P. leopardus* is conserved [71, 72]. The *dmrt* gene family has undergone frequent and independent gene duplication events during evolution. Notably, additional members such as *dmrt6*, *dmrt7*, and *dmrt8* are presence in mammals but are absent in teleost, including *P. leopardus* [73, 74].This highlights a significant evolutionary divergence in the distribution of *dmrt* genes between teleost and mammals.

Based on qPCR, we found that *dmrt* family members showed different expression levels in various tissues of *P. leopardus*. Most *dmrt* genes were highly expressed in the gonads, and the expression levels of *dmrt* gene family members also showed female and male differences at different developmental stages. For example, *dmrt1* has a male-biased expression in the gonads at different developmental stages, and there have been several studies demonstrating that *dmrt1* plays a dominant role in male differentiation in teleost [75]. This is consistent with our results and suggests that *dmrt1* may be involved in spermatogenesis and development in *P. leopardus*. Two paralogs of *dmrt2* (*dmrt2a* and *dmrt2b*), were present in this study, originating from a second round of whole-genome duplication of the ancestral *dmrt2* gene. In many species these two genes have different functions [76]. The results of this study showed that *dmrt2a* was highly expressed only in the gonads as well as in the ovaries at different developmental stages, indicating that *dmrt2a* played an important role in the ovarian development of *P. leopardus*. In contrast, *dmrt2b* was associated with gill and muscle development in fish, which is consistent with our results that *dmrt2b* was highly expressed in gills and muscles of *P. leopardus* without significant differences between males and females at different developmental stages [77]. In addition, other members of the *dmrt* family such as *dmrt3*, *dmrta1* and *dmrta2* show different expression profiles in different fish species. For example, *dmrt3* is highly expressed in the testes of some mammals and fish [78], but not in the testis of *P. leopardus*. *Dmrta1* and *dmrta2* are also expressed in tissues other than gonad, and there are no significant differences between males and females at different developmental stages, suggesting that they may control a wider range of developmental processes, such as neural and embryonic development [69]. These differences in the *dmrt* gene family suggest that the expression patterns and possibly functions of some members in gene family have changed during evolution. Notably, *dmrt1* and *dmrt2a* were only highly expressed in the gonads of the *P. leopardus* and showed a male-female biased expression trend, which was consistent with the results of the *Epinephelus coioides* [79]. During sex reversal in *E. coioides*, *dmrt1* and *dmrt2* expression changed notably in the gonads. This suggests that *dmrt1* and *dmrt2a* may likewise be important for gonadal development and sex differentiation in *P. leopardus*.

Oogenesis is the most important process in female reproduction and determines the hatch quality of fertilised eggs, making oocyte development particularly important. Increasing evidence from teleost has shown that oogenesis is regulated by transcription factors [80–83]. One module related to ovarian development were obtained by WGCNA in this study, among which *dmrt* family member *dmrt2a* in black module is a potential hub gene related *to ovar*ian development. The patterns of *dmrt2a* expression in different species are dissimilar. For example, *dmrt2a* palys an important role in the differentiation of somite in mouse and zebrafish [84, 85]. In bivalves, *dmrt2a* is involved in the differentiation of spermatogenic cells in *Pinctada fucata* and *Chlamys nobilis* [86, 87]. The adult Atlantic cod displayed female-specific *dmrt2a* expression throughout the reproductive season [30]. These differences in *dmrt2a* expression across species suggest that the expression pattern and likely function of *dmrt2a* have changed during evolution. In our study, FISH was used to verify the distribution of *dmrt2a* in the gonad of *P. leopardus*. As a potential oocyte marker gene, *zp4* [62–64] shows co-localization with *dmrt2a*.There was abundant *dmrt2a* mRNA expression in oocytes, suggesting that *dmrt2a* is potentially important for oocyte development in *P. leopardus*. This finding is consistent with studies in *Monopterus albus* where *dmrt2a* signaling was detected in mature oocytes in their ovaries, suggesting a role for *dmrt2a* during oocyte development and maturation [88]. Several studies have shown that *dmrt2a* was considered as a candidate regulator of sexual development like *dmrt1* [89, 90]. *Dmrt2a* acts antagonistically to *dmrt1* in sex determination [91, 92]. *Dmrt2* might be ectopically expressed in the gonadal ridge when *dmrt1* is inactivated, which suggested *dmrt2a* may also have a similar function during sex differentiation [93]. *Dmrt2a* has been demonstrated to regulate primary ovarian differentiation and maintenance in *E. coioides* [79]. In the gonad of *P. leopardus*, we observed a reduction in oocyte number and a significant reduction in *dmrt2a* expression in gonads after *dmrt2a* knockdown. FISH analysis revealed a significant reduction in the fluorescence signal of *dmrt2a* compared to the control, accompanied by the emergence of apoptotic signals. These results suggest that the knockdown of *dmrt2a* induces apoptosis in oocytes, thereby hindering their development. This study highlights the critical regulatory role of *dmrt2a* in oocyte development in *P. leopardus*. However, ovarian development is a highly complex process involving multiple regulatory pathways. Further comprehensive investigations are essential to uncover the precise mechanisms through which *dmrt2a* governs oocyte development.

## Conclusion

In this study, a total of 6 *dmrt* family members were systemically identified and characterized in the Leopard coral grouper (*Plectropomus leopardus*). Expression analysis revealed that *dmrt* genes exhibited divergent expression patterns in different tissues of adult *P. leopardus*. Notably, *dmrt1* and *dmrt2a* were differentially expressed in gonad between male and female, suggesting their potential roles in gonad development and sex differentiation. Furthermore, WGCNA analysis and FISH revealed that *dmrt2a* is predominantly expressed in oocytes and is considered a key factor involved in oocyte development. Knockdown experiments and apoptosis detection demonstrated that *dmrt2a* could regulate oocyte development. In conclusion, our study reveals the potential role of *dmrt2a* in oocyte development in *P. leopardus*, supplementing the understanding of the functional roles of the *dmrt* gene family in vertebrate sex differentiation. These findings provide valuable insights into the mechanisms underlying sex differentiation in teleosts.

## Supplementary Information

Fig S1

Fig S2

Supplementary S1

Table S1

Table S2

## Data Availability

No datasets were generated or analysed during the current study.
