# Supplementary figures and images for: Genome-wide investigation of the dmrt gene family reveals new insight into the gonad development in Plectropomus leopardus: dmrt2a regulate the development of oocytes
Source: Biol Sex Differ. 2025 Oct 29;16:84. doi: 10.1186/s13293-025-00769-6 (PMC12570769; doi:10.1186/s13293-025-00769-6)

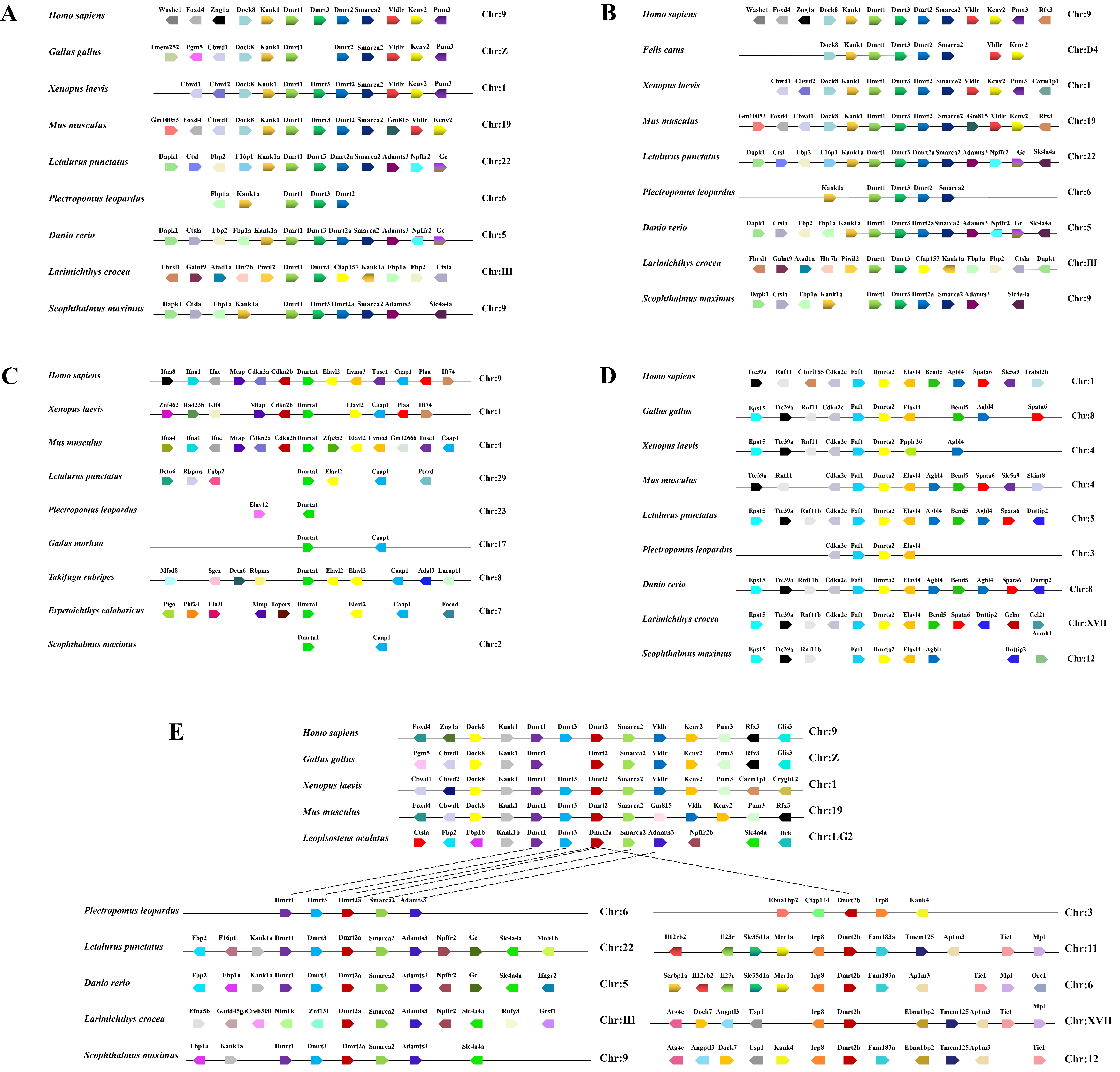

Supplement: Supplementary file 1 — Fig S1 [file 13293_2025_769_MOESM1_ESM.tif]

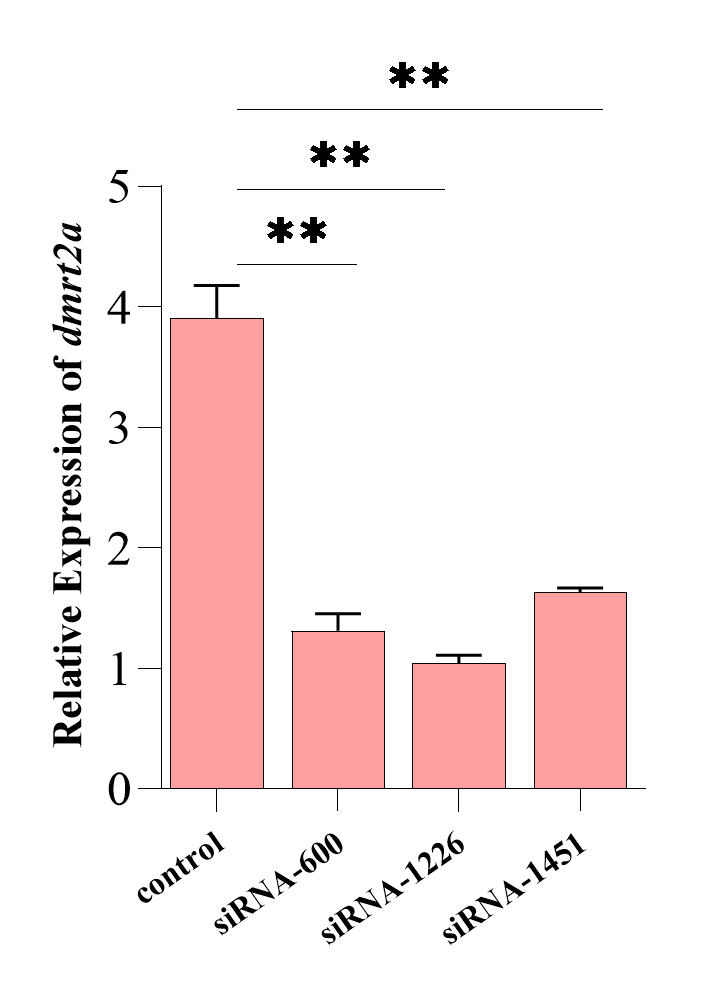

Supplement: Supplementary file 2 — Fig S2 [file 13293_2025_769_MOESM2_ESM.tif]
