# Supplementary material for: Genome-wide investigation of the dmrt gene family reveals new insight into the gonad development in Plectropomus leopardus: dmrt2a regulate the development of oocytes
Source: Biol Sex Differ. 2025 Oct 29;16:84. doi: 10.1186/s13293-025-00769-6 (PMC12570769; doi:10.1186/s13293-025-00769-6)
Supplement: Supplementary file 4 — Table S1 [file 13293_2025_769_MOESM4_ESM.docx]

Table S1. Primer uesd for qRT-PCR.

| Gene | Primers | Sequence (5’-3’) |
| --- | --- | --- |
| *dmrt1* | qPCR-Fw  qPCR-Rv | TCTCCTCTGAAGGGGCACAA  CCGGACTACAAATCCCAAGCT |
| *dmrt2a* | qPCR-Fw  qPCR-Rv | ATCATGCTGGAGCGGGAGT  CGAGTAGGCCGTCTTGTAGTTG |
| *dmrt2b* | qPCR-Fw  qPCR-Rv | TGGAGCAGATCAGGGTATC CTGCTTTGAGGGATCTTGAC |
| *dmrt3* | qPCR-Fw  qPCR-Rv | CACCATACATGACATCAGGAC CTTGCTCTGAAGGTAGGAAAG |
| *dmrta1* | qPCR-Fw  qPCR-Rv | AAACCAAACTCCTGCTACAC GGCTGACGGGATACTTCT |
| *dmrta2* | qPCR-Fw  qPCR-Rv | CACGTATGGACAGGATCAATAA GTGATGGGCTAAGAAGTCATAA |
| *b2m* | qPCR-Fw  qPCR-Rv | GGCAATTCCACCTGACCAAG  CAACCCAGGCATATTCCTTAACT |
