# Supplementary material for: Genome-wide investigation of the dmrt gene family reveals new insight into the gonad development in Plectropomus leopardus: dmrt2a regulate the development of oocytes
Source: Biol Sex Differ. 2025 Oct 29;16:84. doi: 10.1186/s13293-025-00769-6 (PMC12570769; doi:10.1186/s13293-025-00769-6)
Supplement: Supplementary file 5 — Table S2 [file 13293_2025_769_MOESM5_ESM.docx]

Table S2. Primers used for fluorescence in situ hybridization.

| Gene | Primers | Sequence (5’-3’) |
| --- | --- | --- |
| *dmrt1* | ISH-Fw  ISH-Rv | ATTTAGGTGACACTATAGAAGAGGGTGAAGAATGAAGCTGGA  TAATACGACTCACTATAGGGAGAGTCTCAGAGCAGTTGTTGT |
| *dmrt2a* | ISH-Fw  ISH-Rv | ATTTAGGTGACACTATAGAAGAGGGCTTGTAGTCAAATGG  TAATACGACTCACTATAGGGAGAGGGTTAACATCCGTAATC |
| *vasa* | ISH-Fw  ISH-Rv | ATTTAGGTGACACTATAGCTGATTTCCTCGCCGCTT  TAATACGACTCACTATAGGGTGGCTCTTCACACCGTTGTC |
| *zp4* | ISH-Fw  ISH-Rv | ATTTAGGTGACACTATAGCCTGTGTACGTTGAAATC  TAATACGACTCACTATAGGGCTCTCTGTAGCTTCTCTG |
